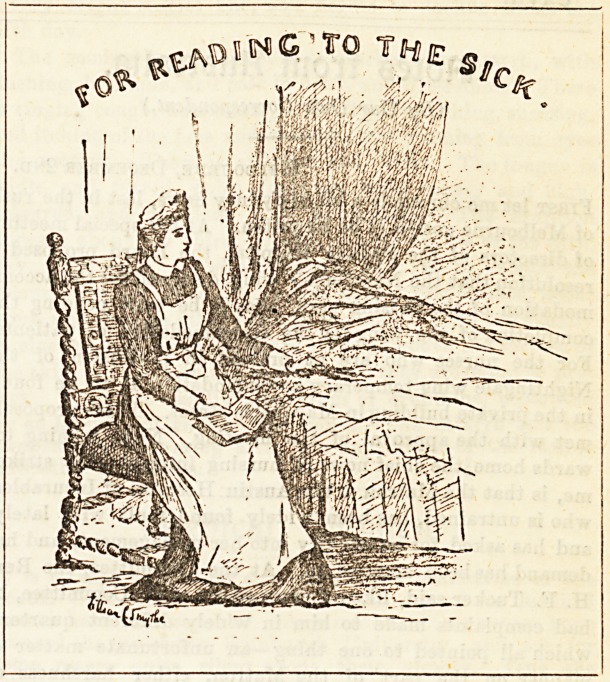# The Hospital Nursing Supplement

**Published:** 1892-02-13

**Authors:** 


### "The Hospital" Nursing Mirror.

Being the Extra Nursing Supplement of "The Hospital" Newspaper.

Contributions for this Supplement should be addressed to the Editor, The Hospital, 140, Strand, London, W.C., and should have the word "Nursing" plainly written in left-hand top corner of the envelope.

### En Passant.

A NEW TRAINING HOME.—Glasgow can boast a new institution which was opened on January 10th by Lady Aberdeen. The principal object of the home is to provide accommodation for ladies during their confinement, or while undergoing special operations. A few female medical cases will also be received, should there be beds available. Any doctor may send in and personally attend his own patients. Associated with the home, but in a separate building, there is a lying-in ward, which has been set apart for poor women, who will be received and attended without fee. The home is solely under the management of Mrs. Hastings, who probably means to make it pay by training monthly nurses. We should have had more confidence in the scheme had there been a committee. Already the home and the nurses have been patronised by 93 city and eight country doctors. At present 12 qualified nurses and six probationers are connected with the home, but the idea is to have 30 qualified nurses and ten probationers, who could take day and night alternately and reside in Bank Street.

HOMES FOR NURSES.—We wrote last week about the necessity for more training homes for nurses—Cumberland Infirmary is just trying to raise £2,000 to build such a home. Here is the Treasurer's report: "When the last meeting of the Committee was held, on the 7th of August, he was able to report that £840 had been promised. Since then he had been promised nearly another £1,000, making over £1,800 altogether. Of that sum he had received, or there had been paid into the bank £1,300. It was suggested by the late Chairman that he should write to certain leading gentlemen in the county for donations. He did so, with the result shown in the second subscription list which had been published. He also wrote to all the railway companies, but he was sorry to say not one had responded favourably to his appeal. The County Hotel Company was the only public body that subscribed, and they gave ten guineas. The rummage sales had been successful, that at Carlisle heading the list with £143, followed by Brampton."

RATE-SUPPORTED HOSPITALS.—We cull the following two items from the columns of a daily paper: "The escape of another patient is reported yesterday from Bradford Fever Hospital, making the second this week. The patient was a girl, sixteen years old, and was found in the early hours of this morning wandering barefooted and clad in a night-dress, about a mile from the hospital. Great indignation is felt in Bradford at the laxity of management at the hospital, which is owned by the corporation, and to which all infectious cases are compulsorily removed." "The Carrick-on-Suir correspondent of the *Irish Times* reports a case in which a shocking state of things in Carrick workhouse was revealed. William M'Grath, an army pensioner and night male nurse, was prosecuted for drunkenness and insubordination. It appeared that he was drunk at night while in charge of a ward of sixteen sick persons, some of whom were in a dying condition. He was sentenced to two months' imprisonment.—A female nurse, in charge of the same ward in the daytime, who left the workhouse at night by scaling the wall, returned in the morning, and was found drunk at her post, was sentenced to fourteen days' imprisonment."

THE ABSENT NURSE.—Again we have to record a sad case of suicide during the absence of the nurse. When will nurses learn that they must never absent themselves from nervous and dangerous cases without leaving someone in charge? The wife of a Mr. Thomas, of Cheltenham, has met with a shocking death. The deceased had been suffering from a nervous malady for some years, and was in charge of a nurse. During the temporary absence of the nurse she appears to have got out of bed, and going up to the third storey threw herself out of the window into the area below, where she was found with her head fearfully crushed, and quite dead.

SHORT ITEMS.—The *National Observer* of January 30th had a charming article on the "Nurses' Co-operation."—H.R.H. the Princess Christian presided at a meeting of the Council of the Royal British Nurses' Association, on the 8th inst., at which it was unanimously resolved that immediate application be made to the Privy Council for the grant of a Royal Charter of Incorporation.—Burton Hospital Saturday Fund have granted £50 to the local Nursing Institution.—The three district nurses of Coventry attended 300 cases last year.—The Buxton district nurse attended 53 cases last year.—Bedworth district nurse attended 143 cases last year.—Nurse Ada Prichler has been speaking on nursing at Croydon.

THE FAMINE IN RUSSIA.—Amongst other pictures of the famine-stricken district of Saratof sent over by Reuter's correspondent, is one of a German nurse. The Zemski Natchalnik had erected a sort of dispensary, which was superintended by a German hospital nurse from Riga, a bright, energetic woman, who was extremely popular among the peasantry. People came from far and near to consult her as to their ailments. Her remedies were of the most simple kind, and her theoretical knowledge was by no means profound; but her natural common-sense and practical kindness were so great that the peasants preferred her to the local doctors. The latter for that reason hated her cordially and denounced her as a witch.

NIGHTINGALE NOTES.—"We Nightingale nurses do not get our full share of your pages, Sir, for we consider ourselves second in importance to no school in the Kingdom. Permit me, then, to inform you that we had a charming concert at St. Thomas's, on Tuesday, which, though it had been delayed, owing to the death of the Duke of Clarence, went off very well. It was, firstly, the Nurses' concert, but there was a good gathering of doctors and students, and friends. The saddest part is to note the absent faces—Sister Christian, who has given up nursing, Sister Albert, who has gone as Matron to Canterbury Hospital, and Dr. Bernays, who used to lecture to us, and who has been claimed by death. The influenza is now almost a thing of the past; I was glad you put a little note saying how well we were cared for during its reign, and I should like to add that our Matron used to come and see us every evening, and sometimes sit quite a long time talking. The patients' entertainments on January 25th and 26th, were two jolly evenings, full of fun and laughter. Altogether we have been very merry and very happy here lately. Do you know that Miss Nightingale sends all her nurses a Christmas card every year? Isn't it good of her? Her interest in this school does not seem to abate in the least, and when any of us get a good appointment she generally sends for us to say 'Good-bye,' and wish us success in our new sphere."

### Lectures on Surgical Ward Work and Nursing.

By Alexander Miles, M.D. (Edin.), F.R.C.S.E.

### Lecture XLIV.—INSTRUMENTS FOR MOUTH.

Tongue Depressors are employed very frequently, and in quite simple procedures. When examining the condition of the fauces or pharynx the tongue is very apt to become

Fig. 1.

arched, and to obstruct the view, and such a depressor as that figured in fig. 1 is used to keep it down. The instrument is of electro-plated metal, consists of two blades of unequal size, united by a hinge joint. Another form of instrument is more convenient when the tongue has to be held down for any length of time, for example, during an opera-

Fig. 2.

Fig. 3.

tion. That by Turch (Fig. 2) answers the purpose very well. When manipulations are being carried out on the mouth and fauces, the finger of the surgeon is apt to be bitten, especially by children, and to prevent this the jointed finger protector (fig. 3) may be worn. This at the same time acts as a gag. For keeping the mouth open during prolonged operations

Fig. 4.

various forms of gag are made use of, for example, that introduced by Fergusson (fig. 4), which is single, and kept at the desired width by means of a screw-button working on a rod. Lister's gag is kept open by a steel ring sliding on the blades. It is double, the two ends being of different sizes. Smith's gag (fig. 6) is a more elaborate instrument, being adapted for both sides of the mouth. In certain classes of patients—*e.g.* alcoholics and lunatics—it is often necessary to forcibly open the mouth, in order, in the first class, to wash out the stomach; in the other, to introduce food. For this purpose the wedge-shaped gag is employed. The edge of the wedge

Fig. 6.

is gently inserted between the teeth, and then by a powerful screw the blades are gradually separated, and the mouth thus opened.

Instruments used in the Operation for Cleft Palate.—The knives used for paring the edges of the cleft in this operation are mounted on long handles, and have a rounded metal stalk, the blade in some being at an angle with

Fig. 8. Fig. 12. Fig. 13. Fig. 14. Fig. 16.

the stalk (Fig. 8), in others straight. Many surgeons use an ordinary bistoury with a good long blade. The needles with which the stitches are passed are all more or less curved. Some have only a single curve, while others are double-curved, and are in pairs, right and left. Raspatories or separators are used to detach the soft tissues from the bone in order that the edges may be approximated

(Fig. 12). Various forms of forceps are employed to seize the tissues as the stitches are being inserted and tightened (Fig. 13). To sponge the parts, the sponge holder, figured in fig. 14, is found very useful. A small sponge is inserted between the toothed blades, and these are approximated by sliding up the ring.

FIG. 15.

INSTRUMENTS USED IN EXCISION OF TONSILS.—This simple operation can quite well be performed with an ordinary pair of vulsellum forceps (Fig. 15) and a curved probe-pointed bistoury, part of the blade of which is protected by a piece of sticking plaster wrapped round it. Some surgeons, however, prefer a knife specially made for the purpose, the proximal end of which does not cut (Fig. 16), and others use a form of tonsil guillotine.

### Inasmuch.

*Lines to a Nurse.—Written by a Patient in Hospital.*

THESE words divine, O kind and gentle Nurse,

The Lord and Master will to thee rehearse;

When all who fight beneath His banner here

Shall round His glorious Judgment-seat appear.

For on thy work the smile of Heaven doth sleep,

And white-robed Angels guardian watches keep;

O'er all thy joys and cares, thy words and ways

The Christ looks down and for thy weal He prays.

Yea, more than this, God's holy courts of song

Resound with praises from His Angel throng.

Hearken, O earth, the very skies are riven

With shouts and songs, 'tis from the mouth of Heaven.

Then onward thro' thy journey on life's way,

God safely guide thee to eternal day;

Away beyond the bright celestial shore,

And then I wish thee well for evermore.

### Presentations.

AT the close of the annual meeting of the Ashton Sick Nursing Association, Miss Bertha Mason, on behalf of the Committee, presented to Miss Thompson, the late Hon. Secretary (who is sailing for Canada), a testimonial, expressing at the same time the hearty good wishes of herself and her colleagues for Miss Thompson's future welfare. The presentation, which takes the form of a carriage clock, is intended to serve as a wedding present, and as a token of the appreciation felt by the Committee for the capable manner in which Miss Thompson has discharged the duties of her office. The clock bears the following inscription: "Presented to Miss Thompson by the Committee of the Ashton-under-Lyne District Nursing Association in recognition of her valuable services as Hon. Sec. January, 1892."

*Acknowledgment.*—Harriet Middleton begs to acknowledge with many thanks, the stamps sent to her on February 5th, in answer to her appeal in that day's HOSPITAL, on behalf of an invalid nurse, by "a nurse," "One of the second thousand."

PATIENCE.

WHAT an easy word to say, but how difficult a grace to make our own. And yet we all have need of patience whether sick or well. In health we pride ourselves on the superiority which makes us "have no patience" with our fellow creatures, while we despise them for their awkward blunders, their helplessness, or their omissions. Well, this is neither a kind nor a healthy frame of mind to encourage, a very bad preparation for the time when we shall ourselves be lying sick and weary with pain, full of discomfort and repining.

Perhaps just now we are feeling annoyed at some slight blunder of an attendant, and instead of making the best of it, we are racking our minds to find out how we can manage to show our anger with the most effect. We are very foolish people. For our own happiness, let us rid ourselves of such unkind thoughts as soon as possible, for they bring in their train, envy, hatred, malice, and all uncharitableness to our neighbours, and discontent and rebellion towards God.

But let us take a step farther and suppose that we are not naturally so very irritable, or else we are trying to get over it, and have determined that impatience shall not master us if we can help it. To ourselves then, we will whisper, "Let him that thinketh he standeth take heed lest he fall." Do not be disheartened, however; mistrust of self is a very wholesome medicine, it keeps us from over boldness, and prevents our having sad disappointments and falls. Besides, how comforting is the remembrance that we have One on our side Who is far stronger than we are, that the trouble is taken off our own shoulders, Who bears our burdens for us, Who in all our afflictions is afflicted, and the angel of Whose presence goes with us in all our way. He is the same Lord who was very pitiful and of tender mercy to Job, whose patience has passed into a proverb. Another loving and faithful saint, Paul the aged, who knew well what numerous and varied trials flesh could endure, bids us not to kick against the pricks, but let patience have her perfect work. But none of these lessons could we learn properly, were it not for the great pattern set by our blessed Lord Himself. On Him we can gaze and never tire, nor fail of gaining help. In Him we shall with patience possess our souls.

O child of God wait patiently,
When dark thy path may be;
And let thy faith lean trustingly
On Him who cares for thee.
And though the clouds hang drearily
Upon the brow of night,
Yet in the morning joy will come,
And fill thy soul with light.

### Notes from Australia.

(*By Our Own Correspondent.*)

Melbourne, December 2nd.

First let me chronicle a bit of Sydney news, lest in the rush of Melbourne gossip it be forgotten. At the special meeting of directors of the Sydney Hospital, the Board proposed a resolution that the Nightingale wing, designed for the accommodation of the nursing staff, should be used pending the completion of the wing for the accommodation of patients. For the nurses who are at present in possession of the Nightingale wing temporary accommodation is to be found in the private building in Macquarie-street. These proposals met with the approval of the meeting. Now, turning towards home the chief news of nursing interest that strikes me, is that the Matron of the Austin Hospital of Incurables, who is untrained, has been widely found fault with lately, and has asked for an enquiry into her management, and her demand has been acceded to. At the Committee, the Rev. H. F. Tucker said, like other members of the Committee, he had complaints made to him in widely different quarters, which all pointed to one thing—an unfortunate matter of manner on the part of the Matron, either harshness or abruptness. So far as her management of the institution was concerned it was admirable. She was a lady possessing a great deal of character and experience, and his inclination was always to regard the Matron with favour. However, he had to say that one matter upon another had been brought under his notice in such a way that he felt there was something radically wrong with the handling of the patients on the part of the Matron. Personally, I fancy the whole mischief arises from the Matron being untrained, and that not her management, but her treatment of the patients individually is to blame. How can an untrained woman know what these poor wretches have to bear? And it is alleged that when she went round the wards with the doctors she would shake her head when a patient was speaking, and then the doctor would pay no attention to what was said. The Committee have already decided that in future the head nurse, who is trained, and not the Matron, shall accompany the doctors on their rounds.

Mrs. Gasterstadt, who for thirteen years has occupied the position of head nurse at the Alfred Hospital, this week resigned her position on account of failing health.

We have just now an Australian Conference on Charity; and the Charities Commission is also sitting. It is all "words, words, words," and the attempt to use philanthropy as a social or political weapon. Men's motives are mostly poor things.

Two ladies, Miss Grace Clara Stone and Miss Margaret White, were last week admitted to the rank and privileges of bachelors of medicine in the University of Melbourne. These ladies are the first who have obtained a diploma in Melbourne. They have completed the five years' course of study, have walked the hospitals, and have passed all the required tests.

You will remember the quaint and charming Cake Fair and Pincushion Contest I described lately, which was given in aid of the District Nursing Society? Well, so successful was the fête that £900 was raised, and the new home for the nurses of the Melbourne District Nursing Society was opened yesterday afternoon by Mr. J. M. Bruce, in the presence of a large gathering of ladies. The home is situated at 66, Cardigan Street, Carlton, and has for its object to provide the nurses with a home where they may be readily found when wanted. Hitherto it has been the practice to give the nurses £100 a year, out of which they found themselves in board and lodging, but this system was found not to work well, because the nurses were not easily accessible. The home consists of one or two terrace houses, and contains accommodation for three nurses and one pupil nurse. Lady Clarke has resigned her position as President of the society, and has gone to Europe.

The Melbourne Hospital Committee have unanimously negatived a proposal to close one or more of the wards, with a view of compelling the Government to assist the hospital management out of its present financial difficulties. There is an overdraft at the bank of £10,000, and an excess of expenditure over an income of £5,000 per annum. An amendment to the effect that a sub-committee be appointed to enquire into the whole subject was carried. Rather than close up any of the wards, it was suggested the Committee, as a last resource, could resign, and leave it to the Government, or to the City Council to carry on the hospital. It is to be hoped that the Committee will like King Stork when they have got him. In another Colonial capital the Government appoints the Chairman and half the Committee of the leading hospital, and the dual control is far from satisfactory.

A remarkable recovery from a pistol shot accident has just taken place in Melbourne. On October 8th a young telegraph operator named Robert Campbell accidentally shot a metal ramrod from a pistol through his left eye, through the brain, and almost through the skull. Dr. Charles Ryan, assisted by Dr. Harris, removed the rod, and last week Campbell left the hospital, quite recovered except for the loss of his eye. Tempting offers have been made to him by showmen to exhibit himself, but he has refused.

An effort called "The Woman's Shilling" is being made to raise £16,000 to add fifty more beds to the Women's Hospital here. If every woman in the colony will give a shilling the sum will be raised. I often fancy we are more original and more successful in begging here than you are at home.

Dr. Gresswell, Medical Officer to the Board of Public Health is trying to get an infectious hospital started, and quite time too. The Wesleyans have opened a nice "Home for Rescued Children" at Cheltenham, holding twenty-five beds. I think this is all the news of interest this month, for we are still all weakly and sad after the influenza.

### Everybody's Opinion.

[*Correspondence on all subjects is invited, but we cannot in any way be responsible for the opinions expressed by our correspondents. No communications can be entertained if the name and address of the correspondent is not given, or unless one side of the paper only be written on.*]

### THE SIR ALEXANDER MORISON PRIZES.

"T. L." writes: In last week's number of The Hospital I saw, under the Presentation Notices, that the late Sir Alexander Morison left two prizes to be given for meritorious attendance on the insane. This announcement is the first I have seen respecting anything of the kind. I think, sir, that in such cases all asylum superintendents should be fully acquainted, and they, in turn, should acquaint the attendants under their charge. I feel persuaded that such things would have a tendency to increase the interest in nursing the insane. I can say, sir, that there are a great number of attendants who are ambitious to gain such prizes, and would, therefore, learn with pleasure that someone had a thought respecting their work. There are certificates of proficiency in mental nursing offered by the Medico-Psychological Association, and I think it very creditable, and worthy of such notice as above mentioned, that so many asylum attendants have, after considerable hard work, deserved and won so many of these certificates since the first examination of last May.

### Examination Questions.

THE prize for January has been accorded to Nurse Jessie Ritchie, of Dundee Royal Infirmary, to whom we have sent Dr. Post's book on "Massage." Second in order of merit stood a tabular answer by Nurse Esther Payne, while the following deserve honourable mention: Nurse Josephine Taylor, E. Fillingham, Sister Maud, Nurse Amy, Nurse E. M. Leake, Nurse Helen Bennett, Nurse Scott, and Nurse E. Ogle. Many sent answers which went far beyond the question asked, and gave miniature medical treatises on fevers generally, and dealt fully with the whole course of the disease down to desquamation in scarlet fever. As we had only asked for the early symptoms, these answers were disqualified. Others wrote neat and concise lists of the early symptoms, but while stating "rash on the second day," never described the character of the rash, or where it first appeared. Every nurse ought to know the early symptoms of small-pox, scarlet fever, and measles, and to be able to distinguish between them, so we are glad to be able to give such a useful account as the following prize answer. The question for February is: "How would you make beef-tea, egg-flip, and peptonized gruel?" Every nurse is eligible for this competition, and a book will be sent to whoever forwards the best recipes. Answers must be written on one side of the paper only, and must bear the writer's full name and address; they must reach this office by February 27th.

### PRIZE ANSWER FOR FEBRUARY.

In SMALLPOX the incubation period is from eight to fourteen days, and the invasion is marked by headaches, rigors, severe pain and weakness in back, sickness and vomiting, drowsiness, and sometimes coma. Sweating is usually present from the first, and is considered an aid to the diagnosis. The tongue is white and furred and the bowels constipated, the pulse full and frequent, and the temperature rising to 104 or 105 at the very onset, and generally reaching to the highest on the third day. The patient is restless, and sometimes delirious. The eruption, which is usually preceded by a scarlet rash on chest, lower part of abdomen, axillæ and groins, appears about forty-eight hours after first occurrence of rigors, but earlier in some cases, and in others after three or four days. It appears as distinct papulæ, about the size of a pin-head, and perceptibly elevated above the surface of the skin, and feeling like small shot under the finger. They come out first on neck, face, and arms, and then on the trunk and lower parts of body. The early appearance of the papules indicates that the attack will be severe, and on the other hand, the late appearance a mild one. The papules become vesicular about the fifth and sixth day. The pain in the back, the sweating, and fall of temperature after the appearance of a rash are the things, amongst the other symptoms, which should lead to a suspicion of smallpox.

SCARLET FEVER.—The time of incubation is about three days, and sometimes very much less. It sets in with the usual symptoms of fever—chilliness and shivering, sickness, with pain in head and limbs. There is thirst, the skin hot and dry, and the temperature going up to 103, or in severe cases to 106, and in mild ones perhaps only reaching 99. The pulse is much increased. Sore throat is complained of, with some difficulty in swallowing, the tonsils, uvula, and soft palate being red and inflamed and covered with white mucus. The papillæ of the tongue project through the white mucus which covers it, constituting white strawberry tongue, or the tongue may be red and inflamed with prominent papillæ (red strawberry). In most cases, but may be sooner, the rash appears the second day, on neck and arms, and then on body and lower limbs, the cheeks being simply flushed. At first the rash appears as numberless red points, which soon unite and become universal over the body. The rash is of a very bright scarlet hue, and begins to decline about the fifth day.

The coming on of MEASLES is marked by catarrh, with flushing, lassitude, and pain in head, and drowsiness. There is ringing cough, hoarseness, difficulty of breathing, sneezing, and itching of the face and watery fluid running from eyes and nostrils. There is sickness and thirst. The tongue is white and furred, pulse frequent, urine scanty and high-coloured, and diarrhœa may be present. The eruption appears about the third, fourth, or even as late as the fifth, day. The spots are small, round, and red in colour, the colour being darker in centre, and appear first on face, neck, arms, and then on other parts of the body. They are usually in clusters, and are not visibly elevated above the surface of the skin, until felt by the finger, when they are found to project. There are usually three crops of eruptions, separated by an interval of twenty-four hours. The catarrh and the temperature increasing after the appearance of the rash is indicative of measles.

### Medical Women.

IT is strange with what nonchalence the ordinances of the Scotch Universities Commissions with regard to medical women have been received. As a matter of fact women have secured the run of the Edinburgh University, and are for some reason quite silent about their victory. True the *Glasgow Herald* says with caution "It is nothing less than a revolution in academic habits which may or may not be beneficial, but which at all events ought not to come unawares upon those interested. The intention clearly is that male and female students shall as far as possible be placed on an equal footing—receive the same training, and acquire all the privileges which the training implies. Now this carries a great deal with it. A graduate as such is entitled to place his name on a list of voters who exercise the Parliamentary franchise. A seat in the University Council would belong of right to a woman graduate and natural claims would hence arise for places in both Court and Senate." But the draft is not yet law and we have waited for some time in vain for the cheers from the female sex; at last week's meeting of the Scottish Association for the Medical Education of Women the ordinance of the Commission was never mentioned. We heard that Misses Balfour, Barclay, Cadell, and Marsh had passed their final examination, and eight other damsels their second examination. The infirmary had offered to set aside forty beds for the use of female students, but it was absolutely necessary for them to have eighty beds to qualify properly. This is all well, but the best bit of news was somehow passed over.

### Notes and Queries.

TO CORRESPONDENTS.—1. Questions or answers may be written on post-cards. 2. Advertisements in disguise are inadmissible. 3. In answering a query please quote the number. 4. A private answer can only be sent in urgent cases, and then a stamped addressed envelope must be enclosed. 5. Every communication must be accompanied by the writer's full name and address, not necessarily for publication. 6. Correspondents are requested to help their fellow nurses by answering such queries as they can.

### Answers.

*Ina.*—Drawings of the prize invalid jackets appeared in our issue for July 11th, 1891.

*Justice.*—Your facts are correct, and we can add the following to your list: (1) A doctor lately threatened to remove his patient because she could not get enough to eat. (2) Some of the nurses are not properly trained. (3) The day and night nurses used to share the same bedrooms. (4) The nurses are often given dry bread for breakfast. Go on; the crash is bound to come soon.

*T. R. S.*—A certificate is given at the end of one year to paying probationers at Guy's Hospital, the Middlesex Hospital, Addensbrooke's Hospital, Cambridge, and others.

*S. R.*—We shall be so glad to use your two little anecdotes for our Hospital Sunday number.

*R.*—(1) It is not necessary to enclose a stamped envelope as a rule. (2) Bucks Infirmary is at Aylesbury—the county town of course. Look it up under Aylesbury in the *Annual*.

*St. Pancras.*—We never pay any attention to anonymous letters.

*E. M. D.*—No hospital worth going to will receive you so young as eighteen. You might apply at the nearest children's hospital, that is your only chance. It is a great mistake to begin nursing before twenty-one years of age.

*E. E. C.*—We cannot print your verses.

### "Australian Papers, Please Copy."

They brought her in late one night; it was a case of so-called pneumonia.

"She've bin a-gettin' wusser all day," snivelled the old woman, her landlady, who had accompanied the patient. "I thought as 'ow the pore thing mightn't last the night, so 'ere she is, for though I let for my livin', as you know, Ma'am, still, I don't hold with no responsibility which it's a thing as a lone widder woman —— "

"There, there!" I broke in impatiently. The cottage hospital of which I was Matron happened to be over-crowded already; my mind was likewise over-crowded with anxieties, so I could not refrain from cutting short the old goody's garrulity.

"Too late for anything!" whispered the house-surgeon, by-and-bye. "It won't be long either!" he added, pitifully.

I have already said the wards were full. The only two nurses I had were on duty with cases they could not possibly leave, so I found I must, myself, undertake the new comer, who made case number fifteen. Besides, the doctor had said there was no hope, and as a rule I watched by the death-beds. After the usual treatment had been gone through, I fancied the patient looked even worse than on her arrival. The fever, however, was abating; but, then, I knew that soon after collapse must set in. The hours went slowly by; I had taken stock of my patient, who lay still and quiet save when the hard, dry cough shook her slight frame. She was a woman no longer quite young; her face was distinctly handsome, but the lines of her mouth were hard, and her straight, dark eyebrows met; her hands, which lay listlessly outside the coverlet, were well-shaped and slim, and on the third finger of the left one two wedding-rings shimmered. Two! I pondered over what might possibly be No. 15's life-history—that she had one I did not doubt. We who see so many phases of human suffering become swift to recognise a living story, bound up in flesh and blood.

"Are you a nurse? Is this a hospital?"

No. 15 was speaking, and rationally, though feebly; the fever had spent itself for the time at least.

"Yes and no!" I answered gently. "This is a hospital, but I am the Matron."

"You're young to be that, aren't you?" were the next slow words. "I, too, was young once," went on the speaker, without a pause, "young and wicked. Yes; jealous, envious, everything that was bad."

I spoke a few quiet, suitable words in answer.

"You mean, then, that I'm going to die?" The question came quickly, and the slim hands, on which the two rings glittered, moved restlessly, but there was no other sign of agitation visible. "To have a claim on that redemption you spoke of just now," went on my patient calmly, "I suppose I must confess. Well, I shall do it—briefly. I was a widow, young, poor, and envious of the happiness of my girl-sister, who was engaged to be married. I planned and plotted to come between Lily and her lover, to gain him for myself. It took time and skill, but little by little I divided them. Then, in her absence I carried him, as from her, a false prayer for release from her promise. Next, I wrote a lying letter to Lily, saying her lover craved to be free, and made me the mouthpiece of his desire. Stung to the heart and hot-headed, they simultaneously broke with each other. Lily stayed away; I've never seen her since. It's easy enough to twist a man to your liking; in time we were married, my sister's lover and I. But he found it all out soon, and cast me off to starve. They say he fled to Australia; anyhow, I gained nothing—nothing."

All this had been brokenly uttered, with many pauses; then there was a silence. The ward was very still; round No. 15 and myself the screen had been drawn; it seemed as if the world were miles away from us two.

"I murdered their happiness," the murmurous voice began again, "deliberately murdered it, and yet you say that for such as I there is pardon and peace! It's not possible. Hush! you don't understand. How should you? Women with calm, meek faces like yours cannot fathom the depths of a heart such as mine. There's one thing, though, you could do for me"—she clutched my wrist. "Listen! Oh, it's no use telling me to stop talking and save my panting breath! Will you do this for me—after—will you send the notice to the newspapers, and add, 'Australian papers, please copy'? He will know then that he is—free, and Lily still lives! It's all I can do."

The murmurs ceased. Between dozing and waking the hours went on, until the morning broke, and the shadows of darkness fled from before the glad sunlight. Then the stillness behind the screen grew more intense. No. 15 lay motionless, but no one watched beside her bed—there was no more need.

### Where to Go.

Both Miss Harrison and Mr. Hewlett's lectures have been put off on account of the epidemic.

Mr. Corney Grain, after a somewhat serious illness, is back at St. George's Hall, and gives daily a most amusing sketch of the vanities and foibles of fancy-dress balls. The 2s. seats in the gallery are excellent. The performance is at three o'clock on Tuesday, Thursday, and Saturday; at eight o'clock on Monday, Wednesday, and Friday.

A second series of free popular lectures were begun at University College on Wednesday evening, when Mr. H. H. S. Cunnynghame lectured on "Taxes and Taxpayers." Among the other arrangements are "History and Literature," by Professor John Nichol; "What is £1?" by Professor H. S. Foxwell; "The Wisdom of the East," by Professor T. W. Ryes Davids; "Ups and Downs of a Mountain Chain," by Professor T. G. Bonney; and "Swift," by Mr. Henry Craik.

The organ recitals at the Albert Hall on Sunday afternoons at half-past three are free to the public, and well worth attending.

The Saturday concerts at the Crystal Palace are resumed to-day, when Mr. Santley is the vocalist. The Electrical Exhibition at present adds to the many attractions of the Palace.

Bach's Passion Music ("St. John") will be sung at St. Anne's, Soho, as usual, every Friday evening at eight o'clock during Lent, and on Good Friday at 4 p.m. The first service will take place on the 4th prox. The organist, Mr. E. H. Thorne, will conduct, and Messrs. Arthur, Charles, and Fairfax Wade, and Messrs. Pinnington and Saunders will take part in the services.

All nurses should go to the Garrick Theatre to see Mr. John Hare as Sir Peter Lund, M.D. There are some delightful hints on medical and nursing matters, and an old Guy's sister appears on the scene.

Venice in London is another place worth a visit; the doors open daily at noon and at six, and a happy three or four hours can easily be spent.

Now we have suggested all sorts of recreation for the month, and we hope nurses will avail themselves of either lectures, concerts, or theatres, for it is good for them sometimes to remember that there are healthy folk on this earth, and that laughter and amusement are not crimes. A nurse is usually far too ready to narrow her life, and therefore her thoughts and sympathies.

## Figures and Tables

**Fig. 1. f1:**
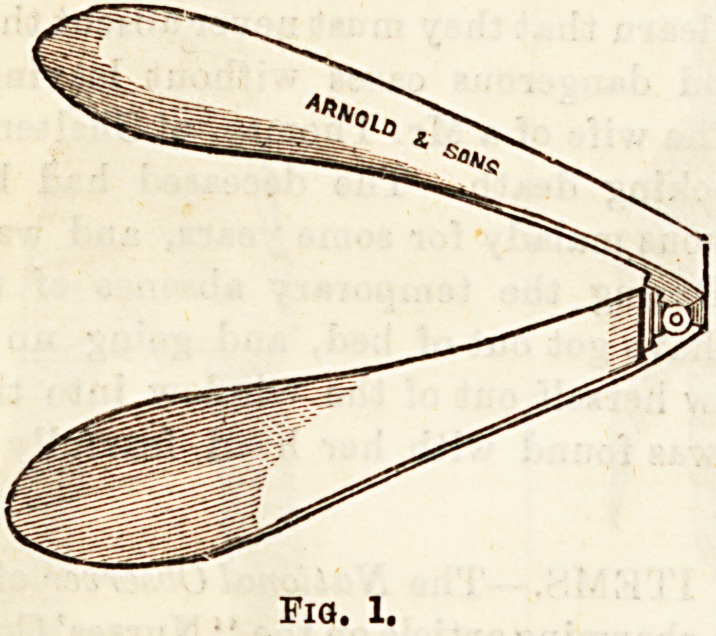


**Fig. 2. f2:**
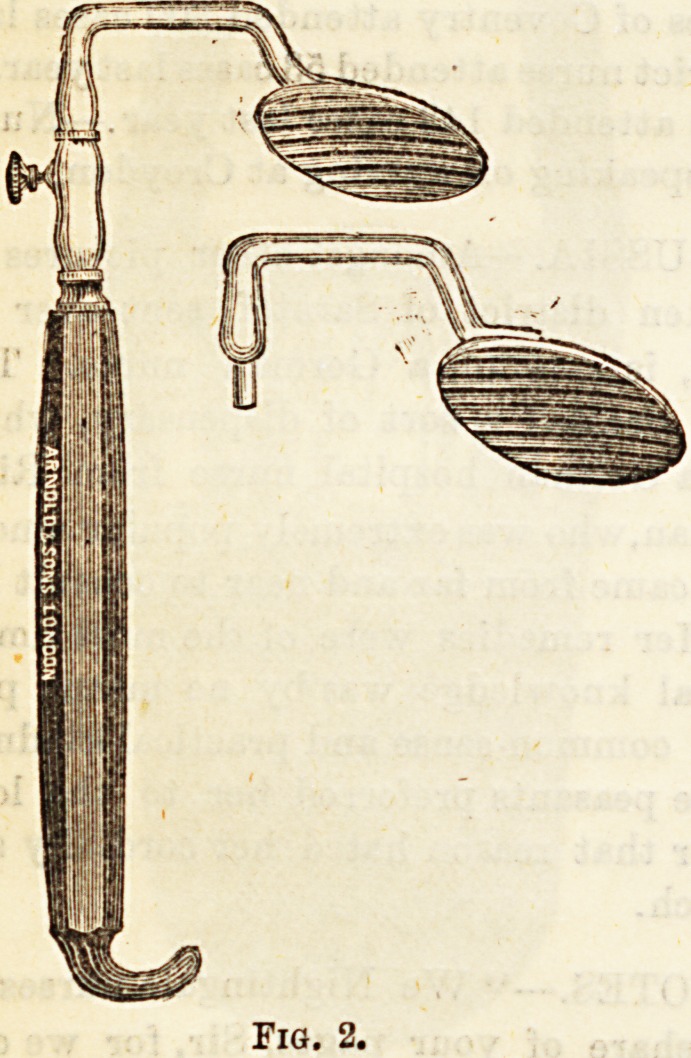


**Fig. 3. f3:**
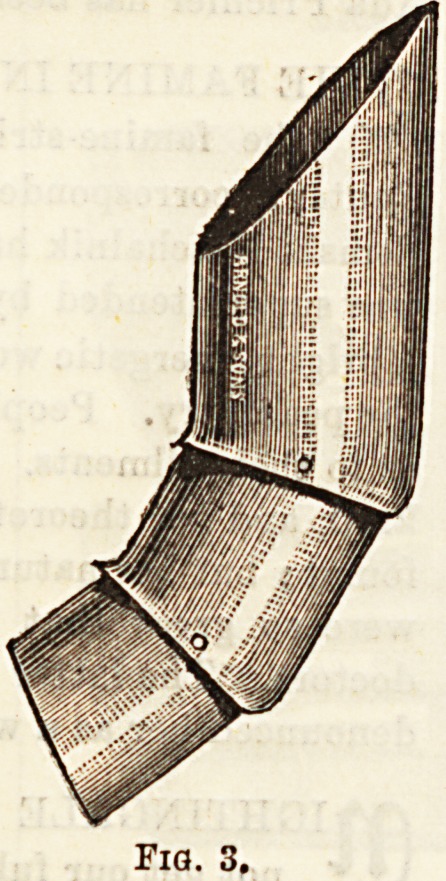


**Fig. 4. f4:**
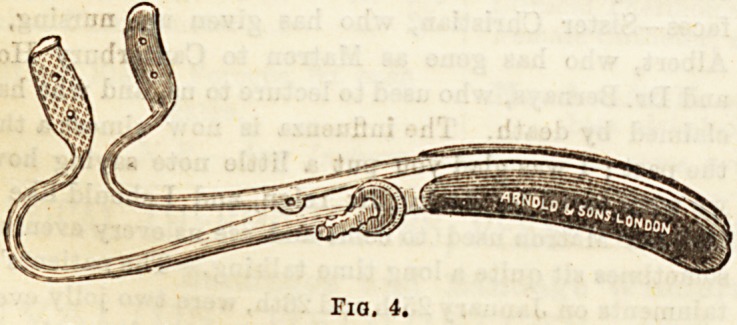


**Fig. 6. f5:**
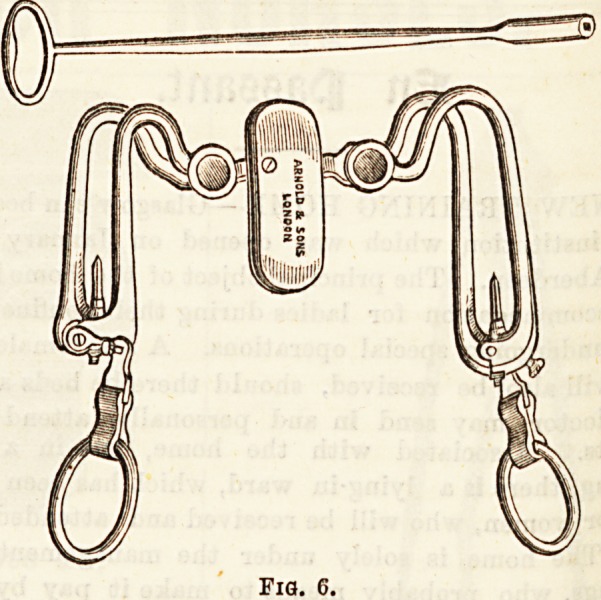


**Fig. 8. f6:**
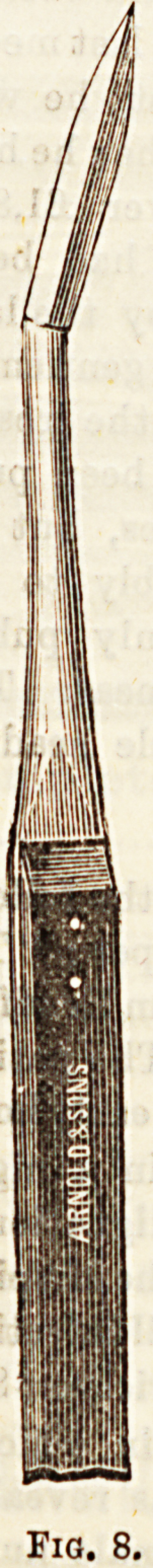


**Fig. 12. f7:**
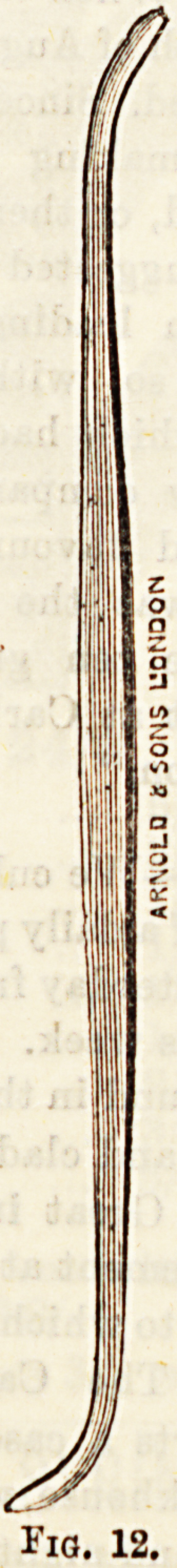


**Fig. 13. f8:**
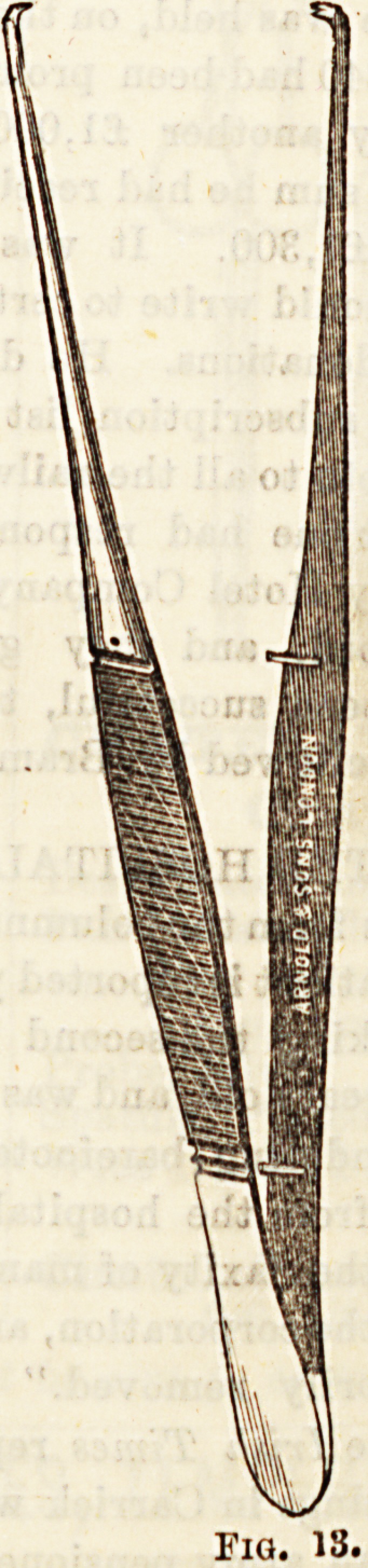


**Fig. 14. f9:**
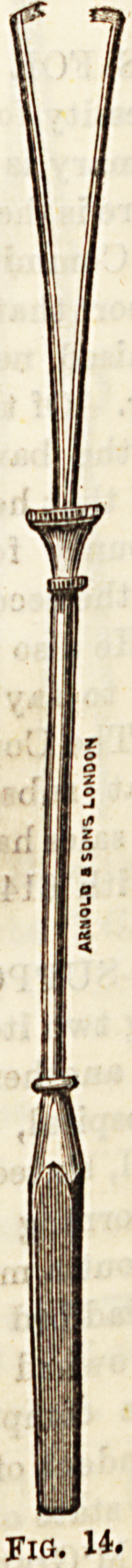


**Fig. 16. f10:**
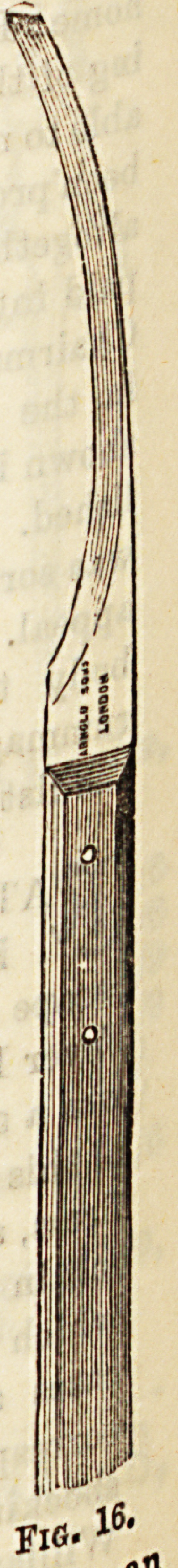


**Fig. 15. f11:**
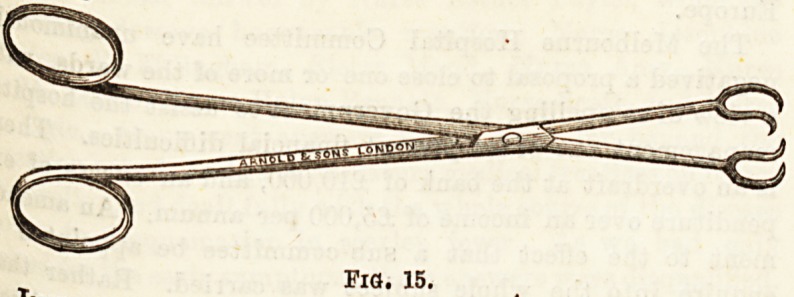


**Figure f12:**